# Sleep disturbance and its associations with severity of dependence, depression and quality of life among heroin-dependent patients: a cross-sectional descriptive study

**DOI:** 10.1186/s13011-017-0101-x

**Published:** 2017-03-20

**Authors:** Vincent Chin-Hung Chen, Hua Ting, Meng-Huan Wu, Tsang-Yaw Lin, Michael Gossop

**Affiliations:** 1 Chang Gung Medical Foundation, Chiayi Chang Gung Memorial Hospital, 613 Chiayi County, Taiwan; 2 Chang Gung University, 333 Tao-Yuan, Taiwan; 3 Department of Physical Medicine and Rehabilitation, Chung-Shan Medical University Hospital, Chung-Shan Medical University, Taichung, Taiwan; 4 Institude of Medicine, Chung-Shan Medical University, Taichung, Taiwan; 5 Ministry of Health and Welfare, Tsaotun Psychiatric Center, 542, No.161, Yu-Pin Rd, Caotun Township, Nan-Tou, Taiwan, Republic of China; 6 King’s College London, Institute of Psychiatry, London, SE5 8AF UK

**Keywords:** Heroin dependence, Sleep disturbance, Depression, Quality of life

## Abstract

**Background:**

Sleep disturbance is common and may adversely affect treatment outcome, mental health, and quality of life in heroin-dependent patients.

Previous studies have focused upon patients receiving treatment. We conducted a cross-sectional descriptive study to explore the 1-month prevalence of sleep disturbance and its associations with socio-demographic, substance-related characteristics, severity of dependence, severity of depression, and quality of life among heroin-dependent patients before entering treatment program.

**Methods:**

The sample (*n* = 514) comprised individuals with heroin dependence attending the methadone maintenance treatment program and the therapeutic community at a psychiatric center in Nantou, Taiwan between 2008 and 2014. Sleep quality was measured using Pittsburgh Sleep Quality Index (PSQI) with a global score greater than 5 indicating sleep disturbance. Centre for Epidemiologic Studies Depression Scale, Severity of Dependence Scale, and World Health Organization Quality of Life-BREF were also approached. T-test, chi-square tests, and multivariate logistic regression were performed to measure associations between variables and sleep disturbance.

**Results:**

The 1-month prevalence of sleep disturbance (PSQI > 5) was 76.3% among 514 subjects with heroin dependence. Heroin users with sleep disturbance had significantly more life events in the previous year, higher rate of unemployment, greater cigarette consumption, more substance related criminal convictions, longer length of heroin use, higher rate of injectors, greater severity of dependence, greater severity of depression, and lower quality of life compared to those without sleep disturbance. Severity of dependence, severity of depression, and physical health domain of quality of life remained significantly associated with sleep disturbance after adjusting for other variables.

**Conclusion:**

Heroin-dependent patients had a high 1-month prevalence of sleep disturbance, and this was associated with greater severity of dependence, greater severity of depression, and poorer physical health-related quality of life. Early assessments and interventions for sleep disturbance among patients with heroin dependence are recommended.

## Background

Heroin dependence, a chronic and relapsing disorder, is a major public health issue for many nations [1]. According to the United Nations Office on Drugs and Crime (UNODC)-World Drug Report in 2015, heroin was the second most widely abused illicit substance [1]. In Taiwan, heroin is categorized as Schedule I drug: it is the most commonly abused drug reported by medical institutions and has significant impacts on health and society [2]. Heroin dependence increases the risk of mortality, drug overdosing, infectious diseases, social problems, criminal behaviors, and psychiatric comorbidity [1–3].

Sleep disturbance is a growing public health concern, with more than one-third of the general adult population suffering from sleep problems [4, 5]. The prevalence of sleep disorder among the Taiwanese general population progressively increased from 10.3 to 46.6% over the last decade [6–8]. Reduced sleep duration and quality is associated with obesity, diabetes, metabolic diseases [9], disability and functional impairment [4]. Sleep disturbance is generally more prevalent in females and increases with age. People with socioeconomic stress, physical illness, and mental disorder are more likely to have sleep problems [10–12]. People with sleep disturbance are also vulnerable to develop psychiatric disorders, especially substance use disorders and depressive disorders [13–15]. The relationship between substance use and sleep disturbance is bidirectional, with sleep disturbance increasing possibility for developing substance use disorders [14, 15], and substance use increasing risk for sleep disorders [16].

Sleep disturbance is common, but frequently under-diagnosed and inadequately managed among heroin-dependent patients. A high prevalence of sleep disturbance, ranging from 70 to 99%, was reported in heroin-dependent patients under methadone maintenance treatment (MMT) [17–20]. Sleep disturbance may adversely affect treatment outcome, mental health, and quality of life in heroin-dependent patients [21–23]. Sleep disturbance is related to heroin abuse, withdrawal from heroin, and methadone detoxification [24]. Factors associated with sleep quality among heroin users were inconsistent in previous studies, and included duration of heroin abuse [17, 20, 25], intravenous heroin use [18], pain [17, 19, 25], depression [17, 20], nicotine dependence [19, 20], and alcohol drinking [20]. Participants in above studies were limited to patients under methadone treatment [17–20], and sleep quality was also affected by dosage and duration of methadone treatment [17, 18, 25]. Less is known about sleep disturbance and related factors among heroin users before treatment. Also, we are not aware of any study exploring the association between sleep disturbance and the severity of dependence (SDS). Furthermore, previous studies had limitations of small sample size.

In this study, we explore the 1-month prevalence of sleep disturbance among patients with heroin dependence before entering treatment program. The association between sleep disturbance and socio-demographic factors, substance-related characteristics, severity of dependence, severity of depression, and quality of life were evaluated in the present study. We hypothesized that heroin-dependent patients with sleep disturbance, when compared with those without sleep disturbance, would have greater severity of depression, greater severity of substance dependence and related problems, and poorer quality of life.

## Methods

### Study design

We conducted a cross-sectional descriptive study to explore the 1-month prevalence of sleep disturbance and its associations with socio-demographic, substance-related characteristics, severity of dependence, severity of depression, and quality of life among patients with heroin dependence.

### Participants and procedures

The inclusion criteria of the study subjects included (A) patients with the diagnosis of heroin dependence based on the Diagnostic and Statistical Manual, Fourth Edition (DSM-IV), and (B) those who entered the methadone maintenance treatment (MMT) program between January 2008 and March 2014 or those who entered the therapeutic community (TC) between May 2011 and March 2014 at Tsaotun Psychiatric Center. Excluded were those who were unable to complete the questionnaire of Pittsburgh Sleep Quality Index (PSQI), for reasons such as illiteracy.

Total 598 patients with heroin dependence enter TC or MMT at Tsaotun Psychiatric Center during study period. After excluding 84 individuals who did not complete the questionnaire of Pittsburgh Sleep Quality Index (PSQI), 514 heroin users participated in this study (483 MMT heroin users and 31 TC heroin users). The number of missing cases was 84 and the response rate was 86.0%. The distribution of age and gender was not different between the missing and observed heroin users.

All participants agreed to participate in this study and provided written informed consent before participation. They were informed of their right to discontinue participation at any time and assurance of confidentiality. The study was reviewed and approved by the Institutional Review Board of the TsaoTun Psychiatric Center, Ministry of Health and Welfare, Taiwan.

### Measurements

All participants completed a set of self-report questionnaires administered by a trained psychiatric nurse on the first day at the treatment program. Questions included age, marriage, employment, years of education, cigarette consumption (number of pack per day), lifetime number of criminal convictions (substance related and non-substance related), age of onset of heroin use, main route of heroin administration (injection or non-injection), and sharing of needles with others (yes or no, life time). Heroin in Taiwan is weighed using a traditional measuring unit: Qian. One Qian is approximately equal to 3.75 g. The frequency and dose of heroin they used per day in the past 30 days before entering treatment were asked.

The following self-administered questionnaires were used in this study:

- The **Pittsburgh Sleep Quality Index (PSQI)** [26] is an effective instrument used to measure sleep disturbance over the last month in adults. Nineteen individual items generate seven “component” scores: subjective sleep quality, sleep latency, sleep duration, habitual sleep efficiency, sleep disturbances, use of sleeping medications, and daytime dysfunction. Each component is scored from 0 to 3, yielding a global PSQI score between 0 and 21, with higher scores indicating a lower quality of sleep. The PSQI has a high reliability and a good validity for people with insomnia, and a global PSQI score greater than 5 indicates “poor sleeper” and clinically meaningful sleep disturbance [26]. The Chinese version of the Pittsburgh Sleep Quality Index (CPSQI) has been confirmed to be reliable and valid using the same cut-off point to determine “poor sleeper” in Taiwan [27].
- The **Centre for Epidemiologic Studies Depression Scale (CES-D)** is a 20-item self-administered scale assessing the severity of depressive symptoms. Subjects are asked how often they experienced each symptom during the past week. Response categories include: (0) rarely or none of the time (less than 1 day), (1) some or a little of the time (l–2 days), (2) occasionally or a moderate amount of the time (3–4 days), or (3) most or all of the time (5–7 days). The Chinese version of the CES-D (CES-D[Ch]) has been confirmed to be a reliable and valid tool for screening and monitoring depressive symptoms in Taiwan [28].
- The **Severity of Dependence Scale (SDS)** is a valid and reliable instrument for measuring the degree of subjective dependence on heroin over the last 12 months [29]. The SDS scale contains five items measuring the main psychological components of dependence, especially compulsive use, which appears to be essential for substance dependence. Each item is scored from 0 (absent) to 3 (severe), yielding a total score between 0 and 15, with higher scores indicating greater severity of dependence. The Chinese version of the Severity of Dependence Scale (SDS[Ch]) has good reliability and validity to measure severity of dependence for heroin users in Taiwan [30].
- The **Family APGAR[Ch] score** (adaptation, partnership, growth, affection, resolve) measures the level of support and communication within the family. Each item is scored from 0 to 3, yielding a total score between 0 and 15, with higher scores indicating poorer family support. The Chinese version of the scale has been validated in Taiwan [31].
- The **Chinese version of the List of Threatening Experiences** (**LTE[Ch]**) is a 12-item scale assessing stressful life events [32]. Three items relevant to society in Taiwan were added to the LTE[Ch]: failing an important examination, serious problems between parents, and serious events related to children [33]. The LTE[Ch] has shown good psychometric properties in Taiwan [33].
- The **CAGE questionnaire** [34] includes four items: Have you ever felt you should Cut down on your drinking? Have people annoyed you by criticizing your drinking? Have you ever felt bad or Guilty about your drinking? Have you ever had a drink first thing in the morning to steady your nerves or to get rid of a hangover (Eye opener)? Item responses on the CAGE are scored 0 or 1, with a higher score an indication of alcohol problems. A total score of 2 or greater is considered clinically significant. Kuo et al. (1999) translated the original CAGE questionnaires to the traditional Chinese version and confirmed the cross-cultural validity of the Chinese version of CAGE questionnaire (CAGE[Ch]) when measuring the alcohol drinking problems in Taiwan [35].
- **The World Health Organization Quality of Life-BREF Taiwan version** (WHOQOL-BREF[TW]) is a valid, reliable and sensitive instrument for the assessment of quality of life in Taiwan [36]. WHOQOL-BREF[TW] contains 28 items classified into the same four domains as the standard WHOQOL-BREF [37]: physical health, psychological domain, social relations, and environment. The first 26 items were the same as the standard WHOQOL-BREF. For the cultural adaptation of the questionnaire to Taiwan, two national items were proposed by patient and expert focus groups after qualitative analysis of the recorded content. The two new items were: being respected/accepted and eating/food. Based on psychometric analyses, the two new national items were classified into social relationships and environment domains, respectively. The WHOQOL-BREF[TW] has been confirmed to be a reliable and valid tool for assessing quality of life in Taiwan [36].

The physical health domain of WHOQOL-BREF[TW] included seven items: Activities of daily living, Dependence on medicinal substances and medical aids, Energy and fatigue, Mobility, Pain and discomfort, Sleep and rest, and Work Capacity. Each item is scored from 1 to 5. The mean score of items within each domain is used to calculate the domain score. Mean scores are then multiplied by 4 in order to make domain scores comparable with the scores used in the WHOQOL-100, yielding a domain score between 4 and 20, with lower scores indicating a poorer quality of life.

The diagnoses of heroin dependence were based on the criteria of Diagnostic and Statistical Manual, Fourth Edition (DSM-IV). Participants were evaluated by trained interviewers using the structured diagnostic interview of Mini-International Neuropsychiatric Interview (M.I.N.I) [38], which has been validated in Taiwan [39], on the first day when they entered TC or MMT.

### Variables

The dependent variable (outcome) was sleep disturbance. Sleep disturbance was defined as a global score of Pittsburgh Sleep Quality Index (PSQI) greater than 5. The independent variables were age, gender, marriage, employment, years of education, life events in the previous year, APGAR[Ch] score, cigarette consumption, CAGE score, criminal convictions (substance related and non-substance related), age of onset of heroin use, length of heroin use, route of heroin administration, dose of heroin, sharing of needles, severity of dependence (SDS), severity of depression (CES-D), and quality of life.

### Statistical analysis

For single variate analysis, t-test for continuous variables and chi-square test for categorical variables were performed to compare the variables between heroin users with and without sleep disturbance. A *p*-value of less than 0.05 was considered to have statistical significance. Multivariate logistic regression model was used to detect the independent factors by including those variables significantly associated with sleep disturbance in the univariate analyses. Analyses were conducted using SPSS 16.0 for Windows.

## Results

### The prevalence of sleep disturbance among heroin users

The socio-demographic and substance-related characteristics and psychiatric diagnoses of the sample are summarized in Table 1. Among 514 heroin-dependent subjects, 76.3% of them exceeded the threshold for clinically meaningful sleep disturbance (PSQI > 5). The 1-month prevalence of sleep disturbance (PSQI > 5) was 75.5 and 82.7%, respectively, for male and female patients with heroin dependence.

**Table 1:** Socio-demographic, substance, and psychiatric characteristics in heroin users

Continuous variables		Number^a^	Mean (SD)
Age (years)		514	36.3 (7.5)
Education (years)		514	9.9 (2.0)
Life events		514	1.8 (2.2)
Family APGAR score		514	5.8 (4.3)
Criminal convictions (substance)		514	2.4 (1.7)
Criminal convictions (nonsubstance)		514	1.4 (1.6)
Onset age of heroin use (years)		514	25.6 (6.7)
Length of heroin use (years)		514	10.7 (5.8)
Dose of heroin (half qian/day)^b^		514	6.5 (6.5)
SDS		514	7.0 (2.7)
CAGE		514	1.4 (1.4)
Cigarette consumption		514	1.2 (0.5)
CES-D		514	21.5 (9.7)
QOL-physical health		514	12.8 (2.1)
QOL-psychological		514	12.1 (2.4)
QOL-social relations		514	12.2 (2.4)
QOL-environment		514	12.1 (2.2)
Global PSQI		514	9.1 (4.3)
Categorical Variables		N	%
Sex			
Male		462	89.9
Female		52	10.1
Marital status			
Single		251	48.8
Married		112	21.8
Divorced, separated, or widowed		151	29.4
Employment	Yes	302	58.8
	No	212	41.2
Injection drug use	Yes	387	75.3
	No	127	24.7
Needle sharing	Yes	44	8.6
	No	470	91.4
Sleep disturbance	Yes (PSQI > 5)	392	76.3
	No (PSQ≦5)	122	23.7

*LTE* List of Threatening Experiences; *APGAR* adaptation, partnership, growth, affection, resolve score; *SDS* Severity of Dependence Scale; *CES*-*D* The center of epidemiological study depression; *PSQI* Pittsburgh Sleep Quality Index; *QOL* The World Health Organization Quality of Life-BREF

^a^Number of total recruited patients = 598, number of missing data = 84. Total 514 participants were analyzed

^b^Half Qian = 1.875 g (number of half Qians of heroin used per day)

### Comparison variables between heroin users with and without sleep disturbance

For single variate analysis (Table 2), heroin users with sleep disturbance (PSQI > 5) had significantly more life events in the previous year, higher rate of unemployment, greater cigarette consumption, more substance related criminal convictions, longer length of heroin use, higher rate of injectors, greater severity of dependence (SDS), greater severity of depression (CES-D), lower quality of life-physical health, lower quality of life- psychological, lower quality of life-social relations, and lower quality of life-environment compared to those without sleep disturbance. These two groups were similar in age, gender, education, marital status, APGAR[Ch] score, non-substance related criminal convictions, dose of heroin, needle sharing, and CAGE score.

**Table 2:** Comparison variables between heroin users with and without sleep disturbance

		Sleep disturbance				t	df	*p*-Value (t-test)
		Yes (PSQI > 5)		No (PSQ≦5)				
Continuous Variables		*N*	Mean (SD)	*N*	Mean (SD)			
Age (years)		392	36.4 (7.5)	122	36.2 (7.5)	−0.32	512	0.751
Education (years)		392	9.9 (2.0)	122	10.0 (1.9)	0.38	512	0.704
Life events		392	1.9 (2.2)	122	1.3 (2.0)	−2.60	512	0.01^*^
Family APGAR score		392	5.9 (4.1)	122	5.3 (4.7)	−1.43	512	0.156
Criminal convictions (substance)		392	2.5 (1.7)	122	2.2 (1.6)	−1.97	512	0.05^*^
Criminal convictions (nonsubstance)		392	1.4 (1.6)	122	1.4 (1.4)	−0.31	512	0.756
Onset age of heroin use (years)		392	25.4 (6.5)	122	26.5 (7.2)	1.64	512	0.102
Length of heroin use (years)		392	11.1 (5.7)	122	9.7 (5.7)	−2.24	512	0.026^*^
Dose of heroin (half qian/day)^a^		392	6.2 (6.3)	122	7.5 (7.3)	1.79	512	0.075
SDS		392	7.5 (2.6)	122	5.5 (2.5)	−7.55	512	<0.001^*^
CAGE		392	1.4 (1.4)	122	1.2 (1.3)	−1.13	512	0.259
Cigarette consumption		392	1.2 (0.6)	122	1.1 (0.4)	−2.70	512	0.007^*^
CES-D		392	23.6 (9.3)	122	14.7 (7.8)	−9.59	512	<0.001^*^
QOL-physical health		392	12.4 (1.9)	122	14.2 (2.2)	8.15	512	<0.001^*^
QOL-psychological		392	11.7 (2.2)	122	13.3 (2.6)	6.42	512	<0.001^*^
QOL-social relations		392	11.9 (2.3)	122	13.1 (2.4)	5.13	512	<0.001^*^
QOL-environment		392	11.9 (2.0)	122	13.0 (2.4)	5.14	512	<0.001^*^
		Sleep disturbance				χ2	df	*p*-Value (Chi-square)
		Yes (PSQI > 5)		No (PSQ≦5)				
Categorical Variables		*N*	%	*N*	%			
Male		349	89.0%	113	92.6%	1.32	1	0.251
Female		43	11.0%	9	7.4%			
Single		185	47.2%	66	54.1%	3.26	2	0.196
Married		84	21.4%	28	23.0%			
Divorced, separated, or widowed		123	31.4%	28	23.0%			
Employed	Yes	219	55.9%	83	68.0%	5.68	1	0.017^*^
	No	173	44.1%	39	32.0%			
Injection drug use	Yes	308	78.6%	79	64.8%	9.55	1	0.002^*^
	No	84	21.4%	43	35.2%			
Needle sharing	Yes	38	9.7%	6	4.9%	2.71	1	0.100
	No	354	90.3%	116	95.1%			

*LTE* List of Threatening Experiences; *APGAR* Adaptation, partnership, growth, affection, resolve score; *SDS* Severity of Dependence Scale; *CES*-*D* Center of epidemiological study depression; *PSQI* Pittsburgh Sleep Quality Index; *QOL* World Health Organization Quality of Life-BREF

^a^Half Qian = 1.875 g (number of half Qians of heroin used per day)

^*^ *p*-Value <0.05

In the Multivariate logistic regression analysis (Table 3), the differences in the SDS[Ch] score (Adjusted OR = 1.19, 95% C.I. = 1.08–1.31), CES-D[Ch] score (Adjusted OR = 1.09, 95% C.I. = 1.05–1.13), and WHOQOL-BREF[TW]-Physical health domain score (Adjusted OR = 0.73, 95% C.I. = 0.61–0.86) among participants with and without sleep disturbance remained significant after adjusting for other variables.

**Table 3:** Multivariate logistic regression analysis for sleep disturbance

	AOR^a,b^	95% C.I.
SDS	1.19	1.08–1.31
CES-D	1.09	1.05–1.13
QOL-physical health	0.73	0.61–0.86

*SDS* Severity of Dependence Scale; *CES*-*D* Center of epidemiological study depression; *QOL* World Health Organization Quality of Life-BREF

^a^Wald chi-square, df = 1

^b^Adjusted Odds Ratio (AOR): Adjusting Life events, employment, cigarette consumption, criminal convictions (substance), length of heroin use, injection drug use, QOL-psychological, QOL-social relations, and QOL-environment

## Discussion

### The prevalence and severity of sleep disturbance

The 1-month prevalence of sleep disturbance (76.3%) among heroin users in this study was within the range of previous studies (70 to 99%) [17–20], and this is higher than that in the Taiwanese general population (10.3 to 46.6%) [6–8]. The mean global PSQI score in the present study was also similar to previous studies [17–20].

### Substance-related variables associated with sleep disturbance

The present study found heroin users with sleep disturbance (PSQI > 5) had significantly greater nicotine consumption, more substance-related criminal convictions, longer duration of heroin use, higher rate of injecting drug use, and greater severity of dependence (SDS) compared to those without sleep disturbance.

Sleep disturbance and significant alterations of sleep architecture are reported during heroin abuse and withdrawal [24]. The underlying mechanisms of heroin-related sleep disturbance may involve decreased adenosine level and activation of arousal nuclei [40]. Previous studies also found length of heroin abuse [17, 20, 25] and intravenous administration of heroin [18] are associated with quality of sleep. However, some variables like the number of substance-related criminal convictions, length of heroin use, and injecting use of heroin diminished significance in the multivariate analysis. The possible reasons were that severity of dependence (SDS) measures core psychological components of heroin dependence and is significantly correlated with the number of substance-related criminal convictions, length of heroin use, and injecting use of heroin [29, 30]. SDS might have much greater effect on sleep disturbance and diminished the significance of these variables in the multivariate analysis.

Sleep disturbance is present both during nicotine consumption and withdrawal. High levels of nicotine cause increased arousal and agitation [41, 42]. Previous studies also found nicotine dependence is related to sleep quality [19, 20]. However, nicotine consumption lost significance in the multivariate analysis of this study. The possible reason was that greater severity of nicotine dependence is found associated with active heroin injection and greater intensity of heroin abuse [41, 42]. The association between nicotine consumption and sleep disturbance might also be weakened after adjusting SDS.

The present study confirmed that severity of dependence (SDS) is an independent risk factor for sleep disturbance. Greater intensity and longer duration of heroin abuse are also reported to be directly associated with lower proportion of slow-wave sleep (SWS) and poorer sleep quality [25]. Further studies are warranted to explore the mechanism between SDS and sleep disturbance. Assessments and interventions for sleep disturbance, like cognitive behavioural therapy for insomnia (CBT-I) [43], among patients with heroin dependence are suggested, especially for those with greater severity of dependence.

### Stress, Depression, and sleep disturbance

Heroin users with sleep disturbance (PSQI > 5) had significantly more life events, higher rate of unemployment, and greater severity of depression (CES-D) compared to those without sleep disturbance.

Psychosocial stressors are reported to activate hypothalamic-pituitary-adrenal (HPA) axis and sympathetic nervous systems and induce sleep disturbance [44]. However, life events and unemployment lost significance in the multivariate analysis of this study. The possible reasons were that stressful life event is also closely associated with increased risk for depression [45]. Prolonged cortisol hypersecretion and activation of HPA axis under high stress conditions have also been speculated to play a role in development of depression [45]. The effect of these stress-related variables on sleep might be smaller than depression. The association between these variables and sleep disturbance might be weakened after adjusting severity of depression (CES-D).

This study indicated severity of depression (CES-D) was an independent risk factor for sleep disturbance. A reciprocal causation and overlapping mechanisms exists between depression and sleep disturbance. The common mechanisms for both conditions may involve hypothalamic-pituitary-adrenal (HPA) hyperactivity, heightened arousal, homeostatic dysregulation, dysregulation of circadian rhythms, impaired neural plasticity, and dysregulation of neurotransmitters [46–48]. HPA hyperactivity has been found to cause hyperarousal and suppress slow-wave sleep (SWS) in both depression and sleep disturbance [46–48]. Further studies are warranted to explore the mechanism between depression and sleep disturbance among heroin users. Assessments and interventions for sleep disturbance are suggested, especially for heroin users with greater severity of depression.

### Quality of life and sleep disturbance

Heroin users with sleep disturbance had significantly lower quality of life (QOL) compared to those without sleep disturbance.

Sleep difficulties were also reported to be related to poor daily function and quality of life [21, 22]. However, QOL-psychological, QOL-social relations, and QOL-environment diminished significance in the multivariate analysis. The possible reasons were that physical illness or impaired daytime activities might also lead to poor perception of quality of life in other aspects. Physical health might have greater effect on sleep disturbance than other perspectives of quality of life and reduce the significance of these variables in the multivariate analysis. On the other hand, it has been reported that severity of depression is closely correlated with diminished quality of life [49, 50]. The significance of these variables could also be weakened after adjusting for severity of depression (CES-D).

Among the variables independently associated with sleep disturbance in the multivariate analysis, QOL-physical health decreases about 27% of risk for sleep disturbance and has the greatest effect on sleep disturbance. Chronic pain and physical illness were also shown to be associated with insomnia and poor sleep quality [10, 17, 19, 25]. The above findings suggest that an intervention for physical health might play an important role in improving sleep among heroin users.

### Limitations and strengths

Our study has a number of limitations. The sample was recruited from a single psychiatric center in Taiwan and the selection of these patients was not random. As a result, there may be selection bias in the study. Furthermore, the study uses a cross-sectional descriptive design, and the study data cannot be used to draw causal conclusions. In addition, the study lacks objective sleep assessment, for instance polysomnography (PSG). Differences between self-rated sleep difficulties and objectively-measured sleep are well recognized clinically. While PSG abnormalities are found to be weakly associated with sleepiness, mental health, and general health [51], the subjectively- perceived sleep disturbance can be an independent predictor of psychosocial outcomes such as worse general health and impaired work performance [52]. Subjectively-measured sleep disturbance is gaining attention and of significant importance in practice [51, 52]. However, the study also has certain important strengths. The present study has relatively large sample size and is the first to explore the independent association between sleep disturbance and severity of dependence (SDS).

## Conclusion

Heroin-dependent patients had a high 1-month prevalence of sleep disturbance, and this was associated with greater severity of dependence, depression, and poorer physical health-related quality of life. Early assessments and interventions for sleep disturbance among patients with heroin dependence are recommended.

## Abbreviations

CES-D: Centre for Epidemiologic Studies Depression Scale
DSM-IV: Diagnostic and Statistical Manual, Fourth Edition
LTE: List of Threatening Experiences
M.I.N.I: Mini-International Neuropsychiatric Interview
MMT: Methadone maintenance treatment
PSQI: Pittsburgh Sleep Quality Index
SDS: Severity of Dependence Scale
TC: Therapeutic community
UNODC: United Nations Office on Drugs and Crime
WHOQOL-BREF: The World Health Organization Quality of Life-BREF
